# Minimally Invasive Adductor Release With Obturator Block for Hip Subluxation in Cerebral Palsy: A Report of Two Cases

**DOI:** 10.7759/cureus.30906

**Published:** 2022-10-31

**Authors:** David A Yngve, Chad L Evans

**Affiliations:** 1 Orthopaedic Surgery and Rehabilitation, University of Texas Medical Branch, Galveston, USA

**Keywords:** obturator, minimally invasive surgery, hip subluxation, cerebral palsy, adductor

## Abstract

Cerebral palsy (CP) is the most common motor disability in childhood and presents with spasticity, increased tone, decreased range of motion, and difficulty with ambulation. Abnormal communication between the cerebrum and the motor fibers leads to functional deficits and long-term adverse sequelae. This case report focuses on hip dysplasia.

Two children with CP who were 4.4 and 3.8 years at initial surgery had substantial hip dysplasia with migration percentages (MPs) by X-ray of 60 and 55 and Gross Motor Functional Classification System (GMFCS) levels of 4 and 5. Each patient underwent minimally invasive selective percutaneous myofascial lengthening (SPML) of the hip adductors and ethanol block of the obturator nerves, along with other indicated procedures. Follow-ups were four and six years for the two cases. Indications for surgery included adductor spasticity with contracture, brisk adductor reflexes, scissoring, and hip dysplasia. The goals were to relieve symptoms and to serve as temporizing measures prior to possible later hip reconstruction.

Results showed that, in each case, the MP improved substantially. Case 1 was a child who initially took steps with assistance and became independent by age six, with GMFCS scores improving from 4 to 2. The MP improved from 60 to 35 over four years. Case 2 was a child of GMFCS 5 who could not stand or take steps. The MP improved from 55 to 25 over six years. In addition to the initial SPML surgery, he had a second SPML surgery 31 months later at age six. This case is noteworthy in that the child consistently used a hip abduction orthosis and an abducted wheelchair through the entire six-year follow-up period.

In conclusion, some young children with a significant hip subluxation can achieve improvement following minimally invasive surgery at medium-term follow-up. Our two children each had special circumstances. One was more highly functioning and became an independent walker. The other had consistent use of a hip abduction orthosis and an abducted wheelchair.

## Introduction

Cerebral palsy (CP) is the most common motor disability in childhood. Hip dislocation due to adductor hyperactivity occurs in up to 35% of patients and is caused by disproportionate spasticity and tone in the hip adductors relative to the abductors. It can result in an increased percentage of uncovering of the femoral head or migration percentage (MP). The onset of this process is the highest in those between the ages of two and five years old with decreased gross motor function [1].

Radiographic studies have proven useful in determining the progression of hip dysplasia along with reliably predicting the probability of future hip dislocation. MPs are significantly greater in those with CP compared to the normal population [2]. The MP is useful in determining prognosis [2-4]. The acetabular index is also valuable, though debate exists as to whether it should be viewed as a supplementary measurement to the MP or whether it stands on its own as a prognostic indicator of hip dysplasia [5-6]. A disadvantage to using the acetabular index (AI) is that, in children with a fixed hip flexion deformity, the resulting anterior pelvic tilt makes the landmarks difficult to determine. 

There are several treatment methods for hip dysplasia in CP, including soft-tissue releases, isolated femoral osteotomy, and combined femoral and pelvic osteotomy. A systematic review revealed mostly retrospective studies with a high degree of bias. There was fair evidence to support combined femoral and pelvic osteotomy, and limited evidence for other treatment methods [7]. However, there is still the opportunity for less invasive soft-tissue release surgery in those up to six years old as a temporizing measure or as a treatment that can have success in some cases, knowing that a more invasive bony reconstructive procedure might be necessary in the future [8-9].

One study demonstrated that open soft-tissue adductor releases of the hips with incisions greater than 1 cm along with phenolization of the obturator nerves can lead to an improved mean MP over a two-year follow-up period, with improved hip abduction range of motion and improved function as measured by the Functional Mobility Scale (FMS). This study included those aged two to six years and only those with initial MP of 45 or less [8]. A study of open soft-tissue releases in 65 children with a mean age of 4.4 years demonstrated good results by MP in 49% with a mean follow-up period of 10.9 years [9]. These studies indicate that open soft-tissue procedures can lead to functional and radiographic hip improvements in a percentage of cases when followed for two to 10 years.

Selective percutaneous myofascial lengthening (SPML) is a minimally invasive surgical procedure with 2-3 mm incisions used to lengthen shortened muscle-tendon units, often combined with ethanol nerve blocks of various nerves when indicated. Structures targeted include the obturator nerve, adductor longus, hamstrings, and the gastrocnemius-soleus complex. Shortened muscle-tendon units have increased tension. This tension can trigger spasticity and dystonia. Lengthening with SPML techniques can lead to improved walking, functional mobility, and quality of life [10]. Such procedures may also reduce hip adductor tension, thus reducing the chronic deforming forces that lead to hip dysplasia.

The percutaneous adductor release may decrease deforming forces on the hip joint. The obturator nerve block with 50% ethanol may also decrease adductor spasticity and tone by directly reducing obturator nerve conductivity. The mechanism might be dissolving an area of the myelin sheath. The obturator nerve block has been reported to have efficacy in patients with increased adductor tone, with reduced pain and spasticity and increased range of motion of the hip joint [11].

To determine whether SPML of the adductor with obturator nerve block might improve hip dysplasia, we present cases of two children who underwent these procedures along with their serial radiographic measures.

## Case presentation

Case 1

This child had a milder case of CP. She was adopted from China at the age of 2.8 years with a history of premature birth. At evaluation at age 4.4, she was able to communicate fully, take steps with assistance, and ambulate with a walker with difficulty. Her Gross Motor Functional Classification System (GMFCS) level was initially 4, and following the procedure was 2. She was able to dress without much difficulty and was able to feed herself with a fork.

A gait exam revealed that, with her father holding both of her hands, her knees brushed each other with every other step, and at times the right leg would get caught behind the left. Knee flexion in mid-stance was 26 degrees on the right and 30 degrees on the left, and ankle equinus in gait was 45 degrees on the right and 40 degrees on the left. Examination on the table revealed hip abduction in flexion limited at 30 degrees on each side. She had brisk reflexes to percussion of the hip adductor and patellar tendons and clonus at the ankles.

**Figure 1:**
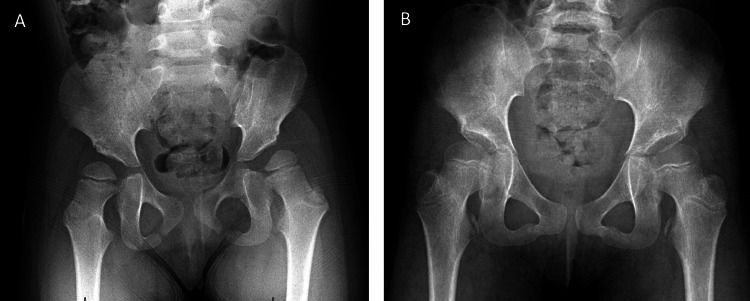
Case 1 X-rays: preoperative (A) and postoperative (B). Gross Motor Function Classification System level 4 initially, later 2. On the preoperative pelvis X-ray, the left hip has a migration percentage (MP) of 60. At four years postoperative, the left hip has an MP of 35.

Her pelvis X-ray showed her left hip to be uncovered, with an MP of 60 (Figure 1A). Consideration was given to SPML with possible bony hip reconstruction in the future.

Her subsequent surgical procedure included bilateral percutaneous multilevel soft-tissue releases at the hip, knee, and ankle levels along with obturator nerve blocks. Specifically, she had releases of the adductor longus and gracilis at the hips; semitendinosus, gracilis, and semimembranosus at the knees; gastrocnemius and soleus at the ankles; and obturator nerve blocks with 2 mL of 50% ethanol. At the four-month follow-up, an examination on the table revealed that hip abduction in flexion was improved to 40 degrees on each side. She no longer had brisk reflexes to percussion of the hip adductors. With her father holding both of her hands, she could take steps with her heels down and there was no scissoring or brushing of the knees.

**Figure 2:**
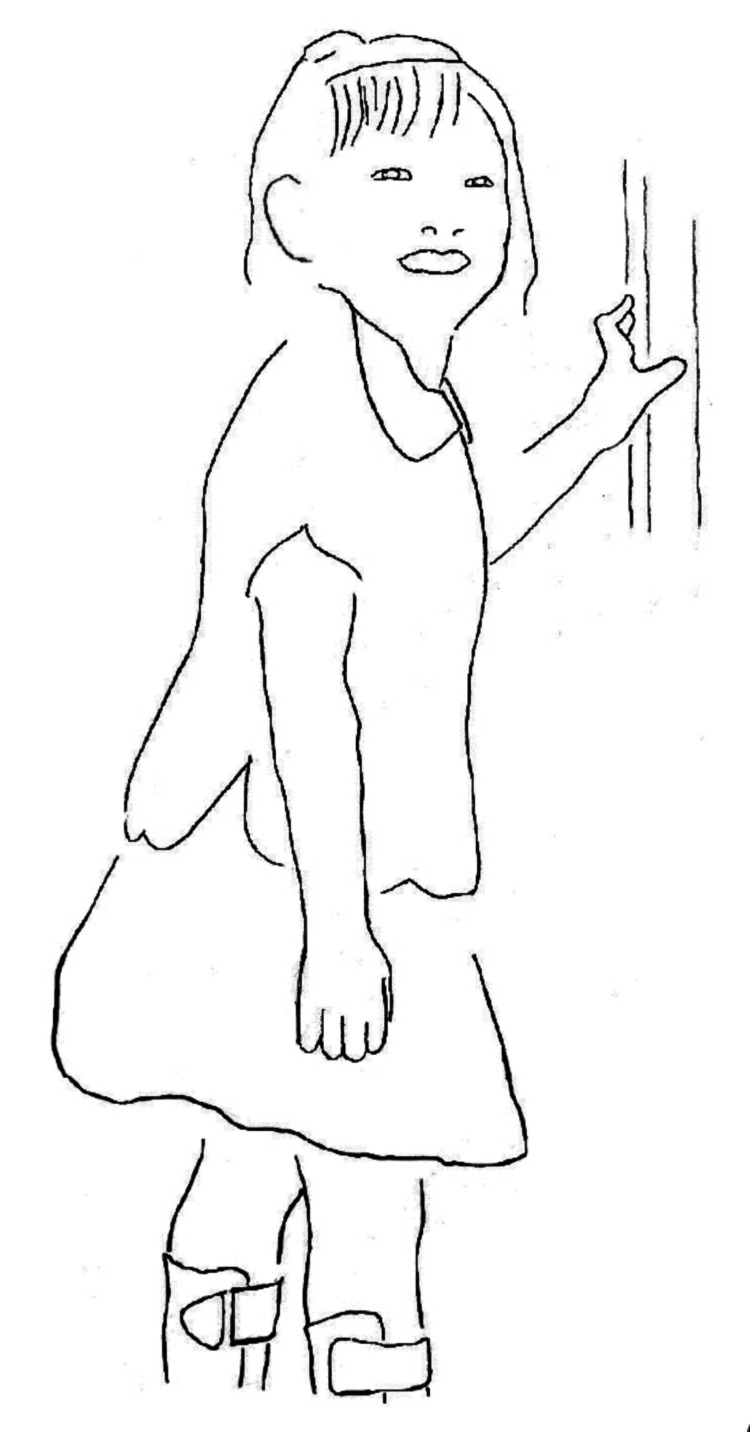
While the patient in Case 1 initially took steps with assistance, her gait improved with time so that she was independent at her four-year follow-up.

Evaluation at four years after the procedure revealed that, in gait, there was moderate hip abduction bilaterally. Follow-up X-rays demonstrated improvement of the left hip MP from 60 to 35 (Figure 1B). The child’s mother described improvements over her previous functionality, and the child walked independently and did not use aids, although she did like to touch walls (Figure 2).

Case 1 discussion

Preoperatively, the MP was 60, which was the highest of the two cases, yet it decreased to 35 over four years of follow-up. In her favor were several factors. She was young at surgery (age 4.4 years) and she was taking steps with assistance with a GMFCS of 4, which made her the higher-functioning child in this series. Furthermore, by two years postoperative (age six), she was walking independently with a GMFCS of 2. Although she had a history of scissoring prior to surgery, it had been decreasing. This suggests a natural history of improving neuromuscular function. However, she did have spasticity of the hip adductors to percussion during preoperative testing. The adductor lengthening and the obturator nerve blocks likely supplemented her neuromuscular improvements. This combination probably led to improvements in hip subluxation.

Case 2

This was the most involved child of the two. He was born a 26-week preemie and sustained a grade IV intraventricular hemorrhage. He subsequently had placement of a ventriculoperitoneal shunt at six months of age. He was discharged from the hospital after 111 days. At the initial evaluation at age 3.8 years, he was nonverbal but could make a few sounds that his mother interpreted as asking for music or an iPad. He could not ambulate, stand, or sit on his own. He was unable to feed himself and could only eat pureed foods. GMFCS level was 5. Seizures were well-controlled with levetiracetam.

Examination of the hips on the table revealed that, with his mother’s assistance in keeping him relaxed, the knees could be separated only 9 inches apart in hip flexion with hip abduction of 5 degrees on each side. Frequently, he was not relaxed, and often the knees were squeezing together. His activities, such as making a sound or reaching or being intent on doing something, would trigger increased tone. The knees had 30-degree flexion contractures. The ankles were in equinus about half of the time, which was when his tone was triggered; otherwise, they could be easily put in a neutral position. He had brisk reflexes to percussion of the hip adductor and patellar tendons and clonus at the ankles.

**Figure 3:**
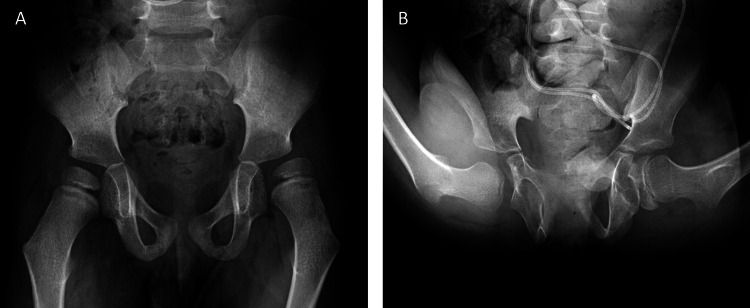
Case 2 X-rays, preoperative (A) and postoperative (B). Gross Motor Function Classification System level 5. On the preoperative pelvis X-ray, the left hip has a migration percentage (MP) of 55. At six years postoperative, the left hip has an MP of 25. The hips were held flexed for the X-ray to compensate for increased lumbar lordosis and to get a good anteroposterior view.

X-ray of his pelvis showed his left hip to have an MP of 55 (Figure 3A). Consideration was given to an SPML procedure with possible bony hip reconstruction in the future.

His subsequent surgical procedure included bilateral percutaneous multilevel soft-tissue releases at the hip, knee, and ankle levels along with obturator nerve blocks. Specifically, he had releases of the bilateral adductor longus and left gracilis at the hips; semitendinosus, gracilis, and semimembranosus at the knees; gastrocnemius at the ankles; and obturator nerve blocks with 3 mL of 50% ethanol.

His mother was very interested in trying to avoid later bony hip reconstruction. The possibility of using a hip abduction orthosis was discussed. She obtained the orthosis a month after the procedures and started using it much of the day and night.

At 31 months postoperative, he returned for a second procedure. He was rolling and creeping, which was new. He was continuing to use the orthosis at night and when on the floor for longer than 15 min, since he tended to scissors when he was on the floor. The wheelchair was adapted for wide hip abduction. It was easy for his mother to get the knees apart. Ankles and knees were doing well following their prior procedures. However, he was developing a windswept posture with left hip adduction. The following day, he had SPML with a left adductor release and a left obturator nerve block with 2 mL of 50% ethanol. The final follow-up at six years following the initial procedure revealed a left hip MP of 25 (Figure 3B).

Case 2 discussion

The MP was 55 initially and it decreased to 25 over six years of follow-up. This is a surprising result for a nonambulatory child of GMFCS 5 following soft-tissue release surgery and nerve blocks. At initial surgery, he was young (age 3.8 years), and young age can allow more hip joint remodeling. At that time, he had strong and persistent scissoring and contractures of the hip adductors with only five degrees of bilateral abduction. There was also spasticity of the hip adductors to percussion. At the four-month follow-up visit, the hip abduction had improved to 45 degrees on each side and there was no spasticity to percussion of the hip adductors, indicating a good effect from the adductor releases and obturator nerve blocks. At the 31-month follow-up visit, the right hip abduction had decreased to 35 degrees, but the affected left hip abduction had decreased to 15 degrees. There was again spasticity to percussion of the bilateral hip adductors. His second procedure at that time addressed the left hip adduction and spasticity and was likely another positive factor in his good hip outcome at the final follow-up.

**Figure 4:**
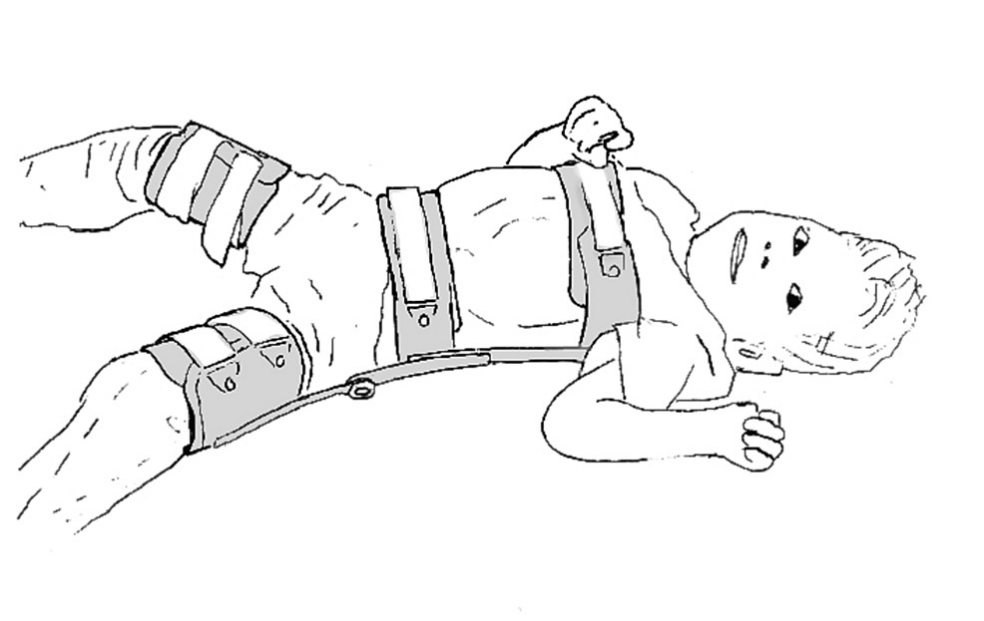
The family of the patient in Case 2 chose to use a hip orthosis in the hopes that they could avoid hip reconstructive surgery. The orthosis was worn part-time every day starting one month after the initial procedure, through the six-year follow-up, and was well tolerated.

**Figure 5:**
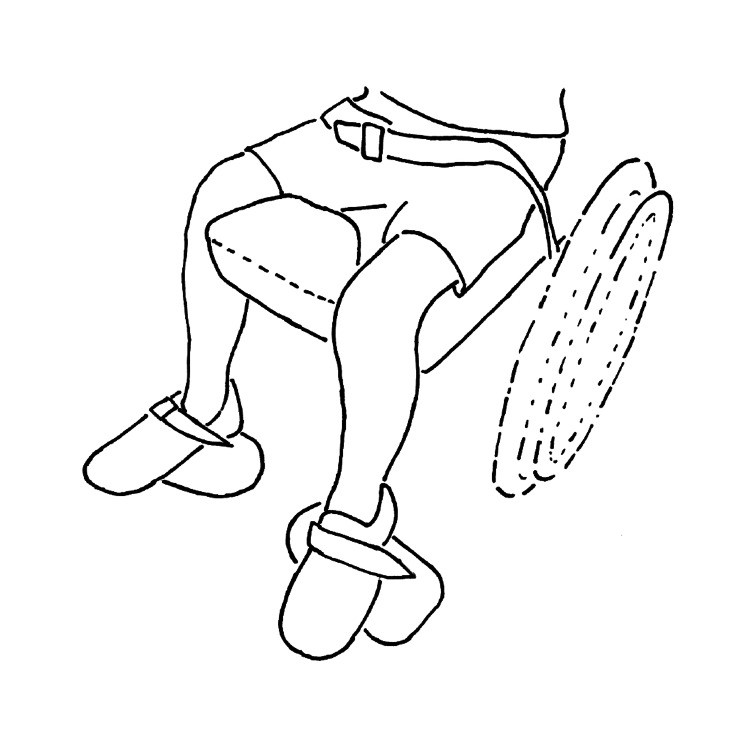
The family of the patient in Case 2 had the wheelchair modified for hip abduction. Wheelchair modifications included a large pommel to separate the knees and rotation of the foot plates outward for increased space between the feet.

The hip orthosis was well tolerated with no pressure sores (Figure 4). His mother reported that he developed an “insignificant callous at the lower back.” She also reported that the hip orthosis was easier than the knee immobilizers following the hamstring releases. She felt that the hip orthosis would have been painful for him prior to the release due to his adductor tightness, but after the release, he was loose enough to be comfortable in the brace. After about two years of wear, the orthosis was adjusted by bending the metal pelvic band wider and attaching a longer pelvic strap. After about four years of wear, he received a new orthosis due to growth. In general, adjustments were less than yearly, and less often than his ankle-foot orthosis adjustments. In addition to the hip orthosis, the family was interested in an abducted wheelchair [12]. They took this concept to their wheelchair provider and had custom modifications made (Figure 5).

## Discussion

Adductor hyperactivity in CP presents with both initial functional problems and the risk of late hip dislocation, which is often a painful and debilitating condition that can further decrease the quality of life. SPML with obturator nerve block is an attempt to reduce both the early and late aspects associated with adductor hyperactivity. In these two cases, the early manifestations were well managed by the procedures, with both patients demonstrating increased hip abduction range of motion and decreased hip adductor spasticity. The parents of both children were satisfied with the early results. For late aspects, we must assess the MP.

**Figure 6:**
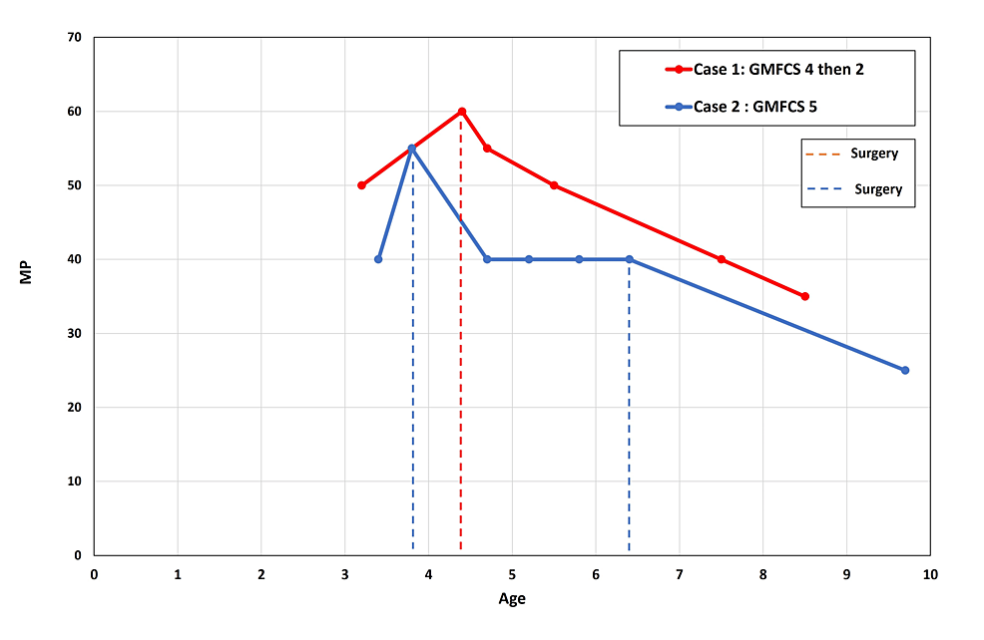
Graph showing migration percentage of Case 1 and Case 2, initially and through four and six years of follow-up.

The MP is subject to intraobserver and interobserver variation. Studies have found both intraobserver and interobserver variations of the MP are 3%-5% [13-15]. Thus, given that the MP measurements in this study were all performed by one individual, a change of the MP of more than 5% by our measurements would indicate that the change is real (Figure 6).

The two cases in this report had adductor releases and obturator nerve blocks which were augmented by external factors. One child was higher functioning and developed into an independent ambulator. The other had prolonged hip abduction bracing with an abduction orthosis and with an abducted wheelchair.

Other reports describe external factors as well. In one study, 16 children were followed for two years. The procedures were open adductor longus and gracilis release with open phenol application to the anterior branch of the obturator nerve along with botulinum toxin to the calves and hamstrings. External factors included the exclusion of GMFCS 5 children and the use of abducted long-leg casts for three weeks followed by hip abduction bracing at night for six months. Increased physical therapy was recommended for six months [8].

In another study, 65 children were followed for a mean of 10.8 years. The procedures were open adductor longus and gracilis release with partial adductor brevis release along with psoas release. Non-walkers had neurectomy of the anterior branch of the obturator nerve. Proximal hamstring releases were performed when needed to treat a popliteal angle greater than 45 degrees. External factors included the use of knee immobilizers for 8-12 h a day for one month along with a physical therapy prescription for sessions three times a week for six weeks. A prone position in bed for at least half the time was encouraged, as was stretching of the adductors daily. Results were good in 49%, at a mean follow-up of 10.8 years [9].

The overall pattern here is that soft-tissue release is a modality that has effectiveness in some children in the treatment of hip subluxation. Orthotic use, as well as recommendations for physical therapy, are common. For families trying to avoid a larger osteotomy surgery, these treatment methods can be recommended as temporizing measures that can sometimes prevent future osteotomy surgery.

## Conclusions

This is a report of two cases of children who underwent minimally invasive soft-tissue lengthening with SPML techniques along with ethanol obturator nerve blocks. The children were 4.4 and 3.8 years at the time of the initial procedures. Both showed major improvement in the MP. In each case, there was an improvement in hip subluxation in the medium term. It is known that open soft-tissue releases are associated with improvement in hip subluxation in some cases. We document that percutaneous soft-tissue releases along with ethanol obturator nerve blocks can be associated with improvement in hip subluxation. When this occurs, often other favorable circumstances can be identified. In our cases, those were a higher functioning GMFCS 4 and later 2 in one case and prolonged hip abduction orthotic and abducted wheelchair use in another.
